# Supplementary material for: Telomere as a Therapeutic Target in Dedifferentiated Liposarcoma
Source: Cancers (Basel). 2022 May 25;14(11):2624. doi: 10.3390/cancers14112624 (PMC9179266; doi:10.3390/cancers14112624)
Supplement: Supplementary file 1 [file cancers-14-02624-s001.zip › Figure S1.pdf]

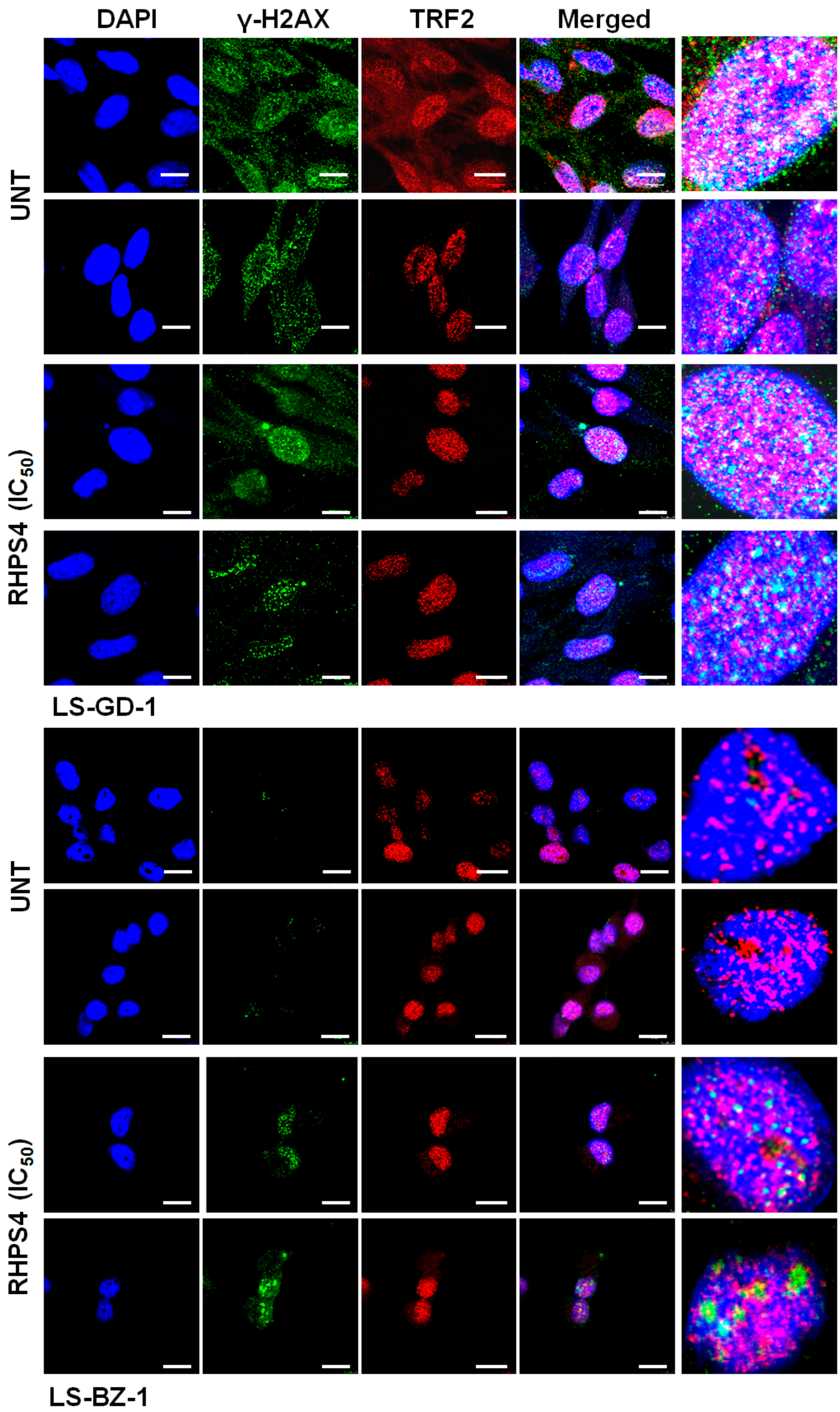

Figure S1. DDLPS cells exposed to RHPS4 are characterized by DNA damage. Representative photomicrographs showing untreated and RHPS4-treated DDLPS cells probed with anti-γ-H2AX (green signal) and anti-TRF2 (red signal) antibodies. Nuclei were counterstained with DAPI (blue). Magnification

×63, scale bars:10 μm. The zoomed images on the right highlights the co-localization signal between γ-H2AX and TRF2 (yellow).
