# Supplementary material for: Telomere as a Therapeutic Target in Dedifferentiated Liposarcoma
Source: Cancers (Basel). 2022 May 25;14(11):2624. doi: 10.3390/cancers14112624 (PMC9179266; doi:10.3390/cancers14112624)
Supplement: Supplementary file 1 [file cancers-14-02624-s001.zip › Figure S2.pdf]

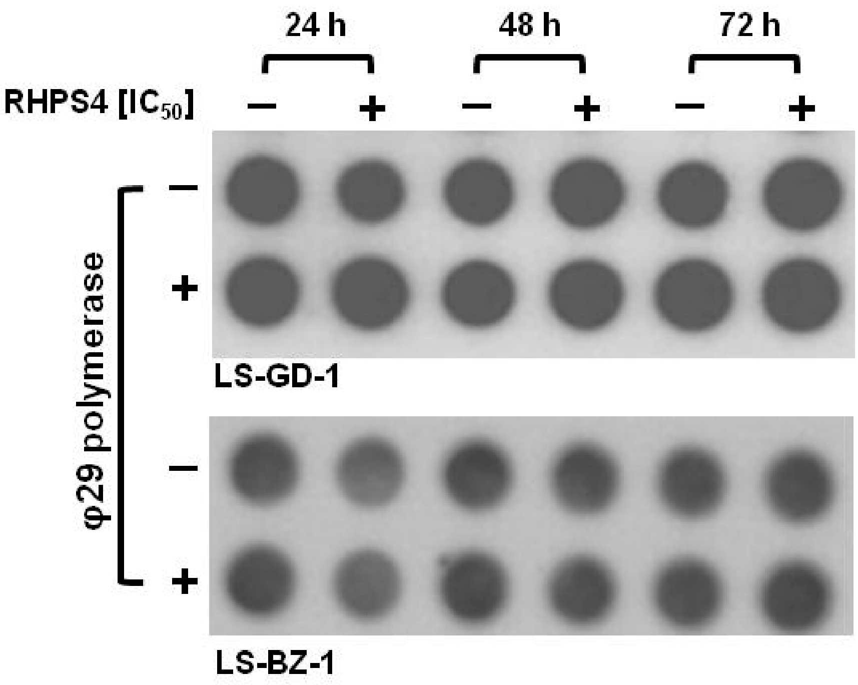

Figure S2. Hybridization of a CCA dot blot with an Alu probe. Representative image of a dot blot showing the re-probing of a CCA filter with a ^{32}P-labelled Alu probe to ensure for equal sample loading.
