# Supplementary material for: Telomere as a Therapeutic Target in Dedifferentiated Liposarcoma
Source: Cancers (Basel). 2022 May 25;14(11):2624. doi: 10.3390/cancers14112624 (PMC9179266; doi:10.3390/cancers14112624)
Supplement: Supplementary file 1 [file cancers-14-02624-s001.zip › Figure S3.pdf]

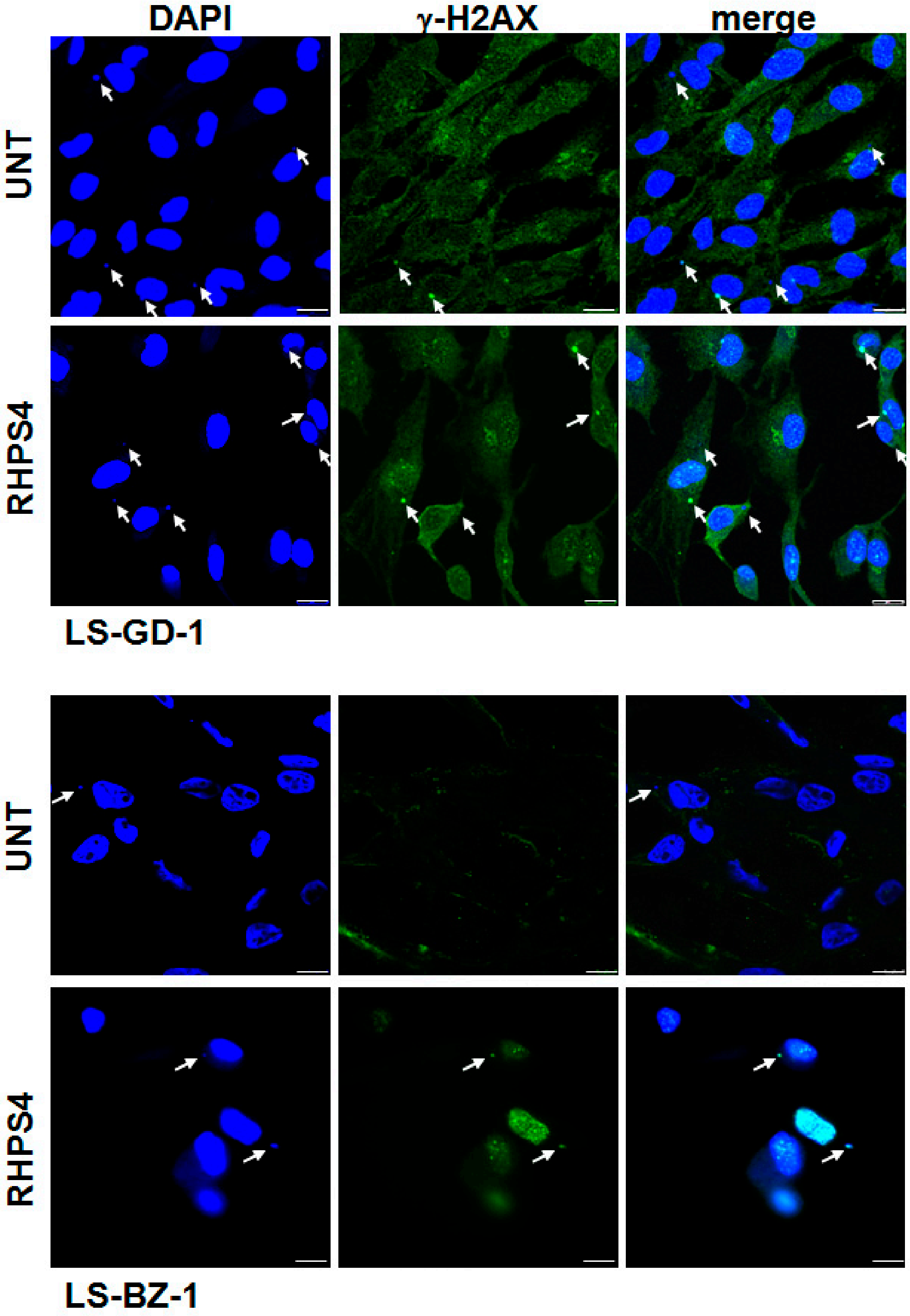

Figure S3. DDLPS cells exposed to RHPS4 are characterized by micronuclei accumulation. Representative immunofluorescence photomicrographs of RHPS4-treated and untreated LS-GD-1 and LS-BZ-1 cells probed with an anti-γ-H2AX antibody (green). Nuclei were counterstained with DAPI (blue). White arrowheads indicate DAPI- and γ-H2AX-stained micronuclei. Magnification ×40; Scale bar: 20 µm.
