# Supplementary material for: Telomere as a Therapeutic Target in Dedifferentiated Liposarcoma
Source: Cancers (Basel). 2022 May 25;14(11):2624. doi: 10.3390/cancers14112624 (PMC9179266; doi:10.3390/cancers14112624)
Supplement: Supplementary file 1 [file cancers-14-02624-s001.zip › File S1.pdf]

Supplementary Information File

Original, uncropped blots and gels

Manuscript title:

Telomere as a Therapeutic Target in Dedifferentiated Liposarcoma

Irene Alessandrini[1], Stefano Percio[1], Eisa Naghshineh[1], Valentina Zuco[1] , Silvia Stacchiotti[2], Alessandro Gronchi[1], Sandro Pasquali[1,3], Nadia Zaffaroni[1*,†], Marco Folini[1*,†]

[1] Molecular Pharmacology Unit, Department of Applied Research and Technological Development, Fondazione IRCCS Istituto Nazionale dei Tumori di Milano, Via G.A. Amadeo 42, 20133 Milan, Italy;
[2] Adult Mesenchymal Tumor and Rare Cancer Unit, Department of Cancer Medicine, Fondazione IRCCS Istituto Nazionale Tumori, via Venezian 1, 20133, Milan, Italy;
[3] Sarcoma Service, Department of Surgery, Fondazione IRCCS Istituto Nazionale Tumori, via Venezian 1, 20133, Milan, Italy;
* Correspondence: nadia.zaffaroni@istitutotumori.mi.it; marco.folini@istitutotumori.mi.it; Tel.: +39-022-390-3260 (N.Z.); +39-022-390-5027 (M.F.).

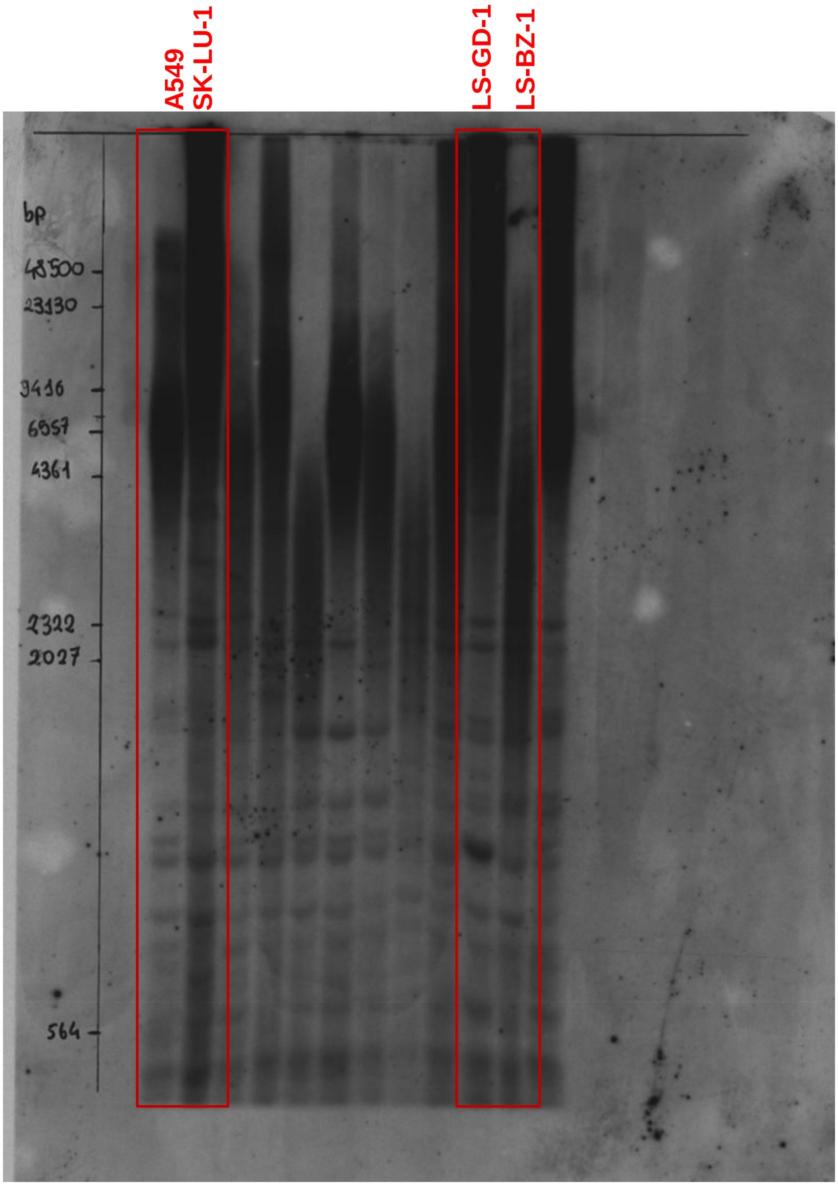

Figure S4. Full Southern blot of Figure 2 (A)

First replicate

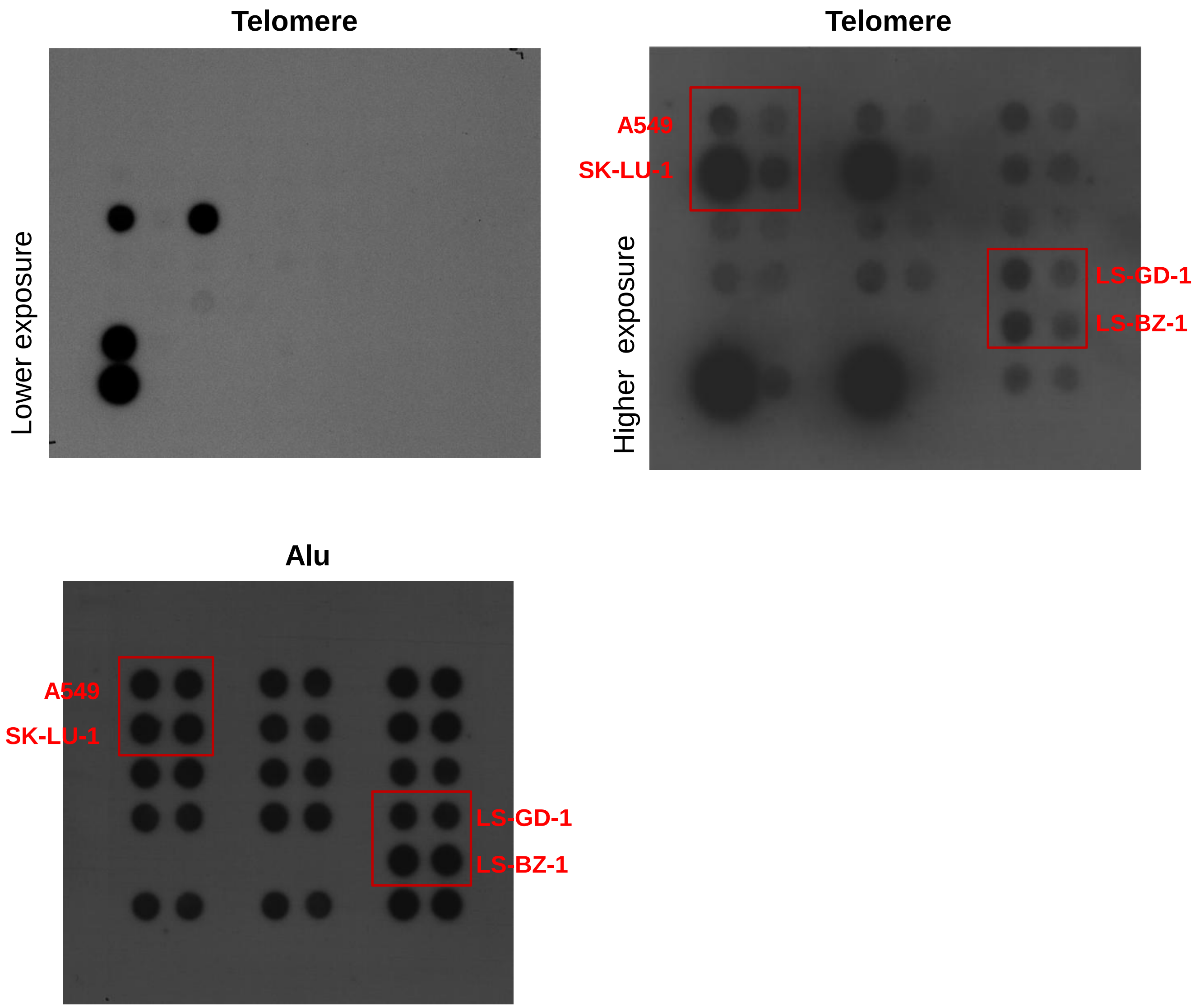

Figure S5. Full Dot Blot of Figure 2 (B).

Second replicate

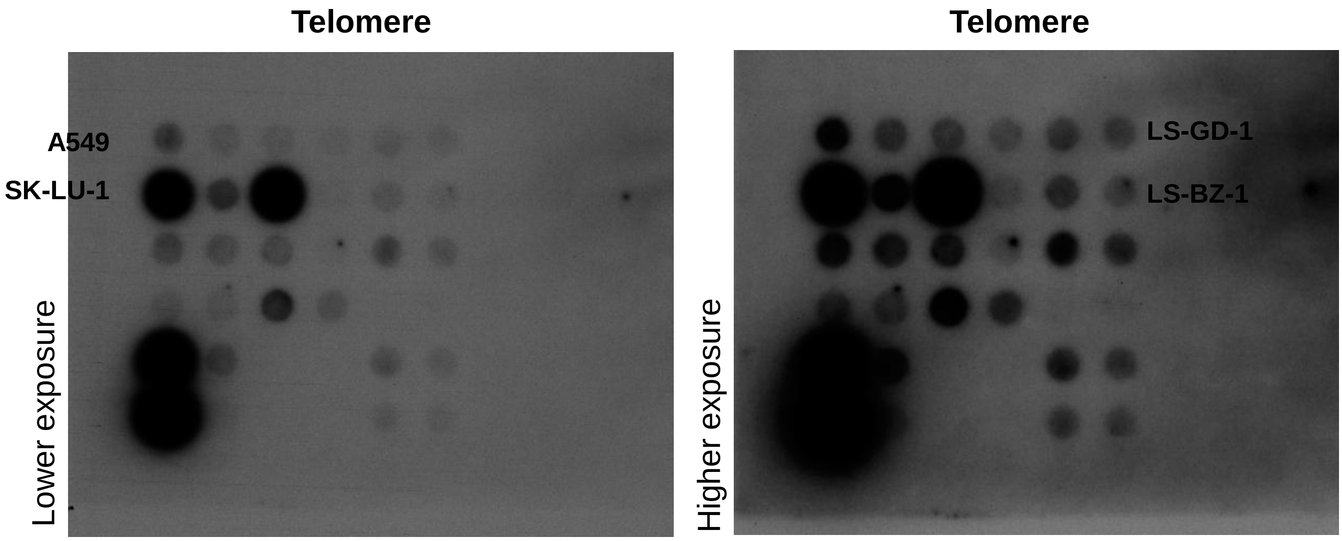

Third replicate

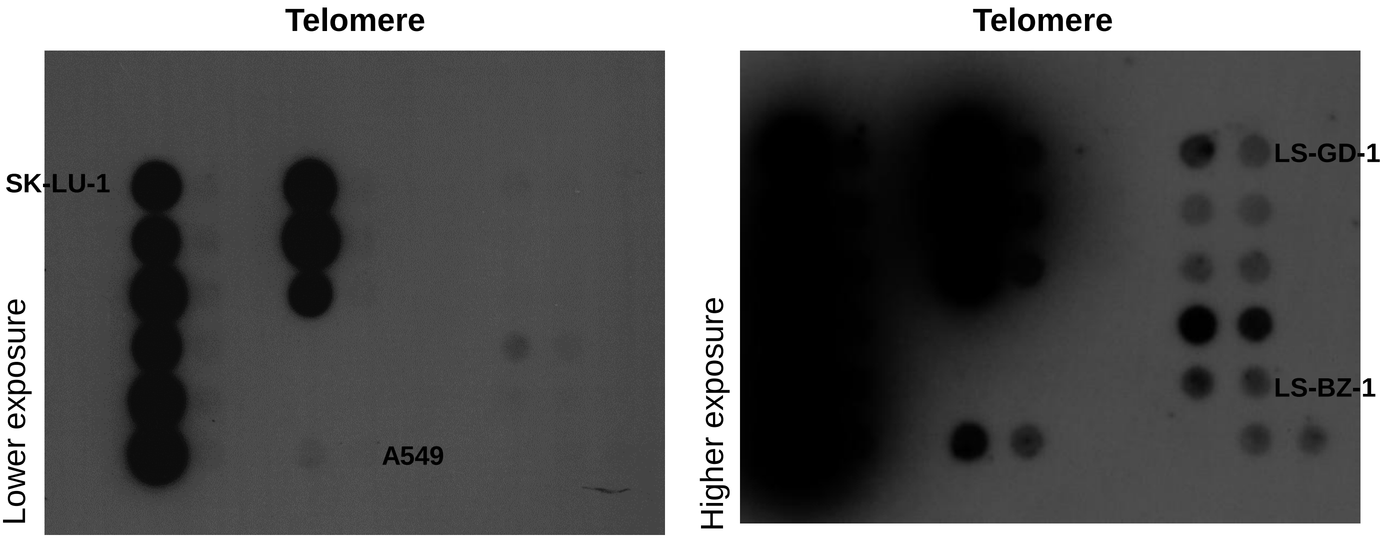

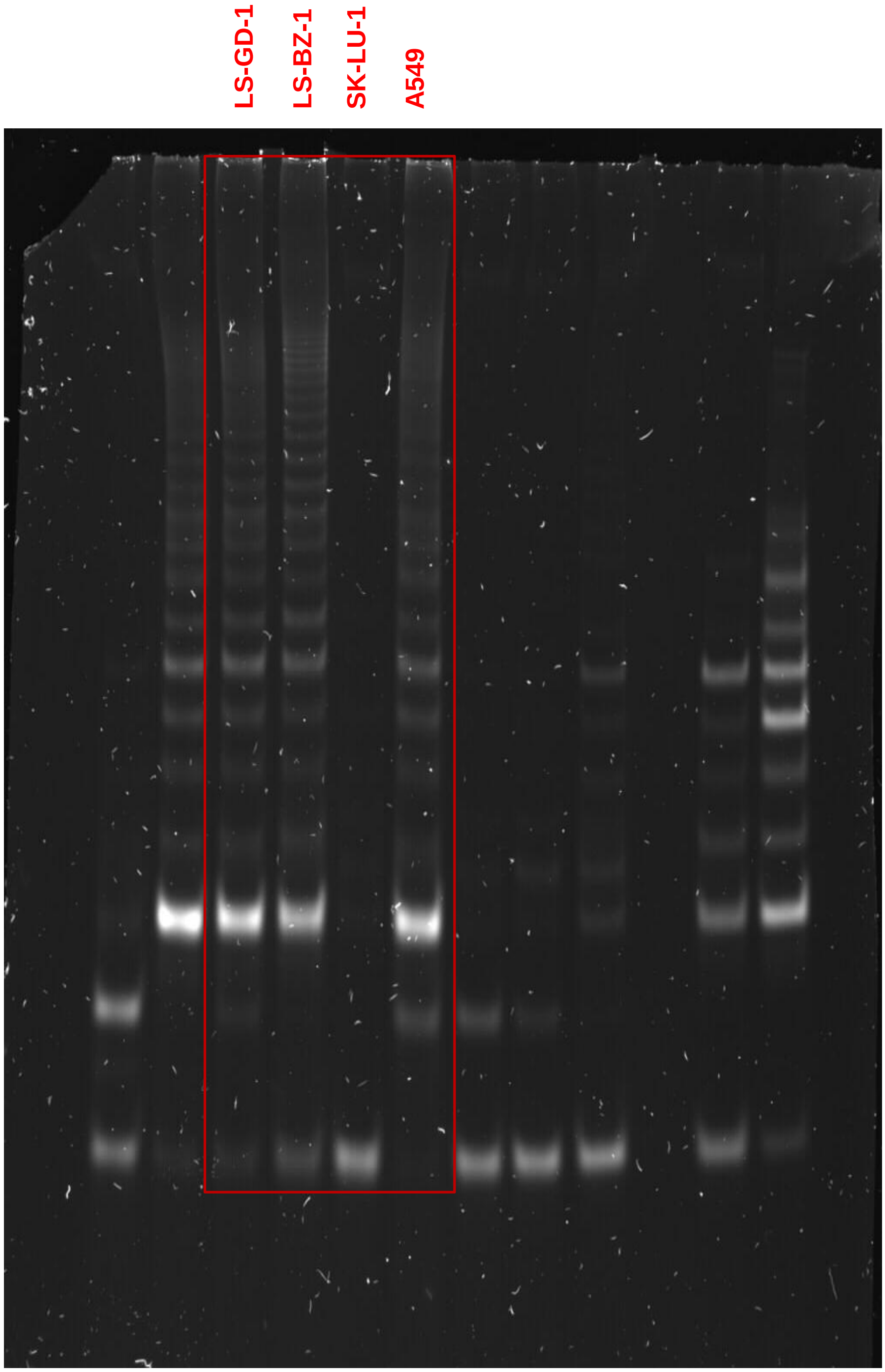

Figure S6. Full gel of Figure 2 (D).

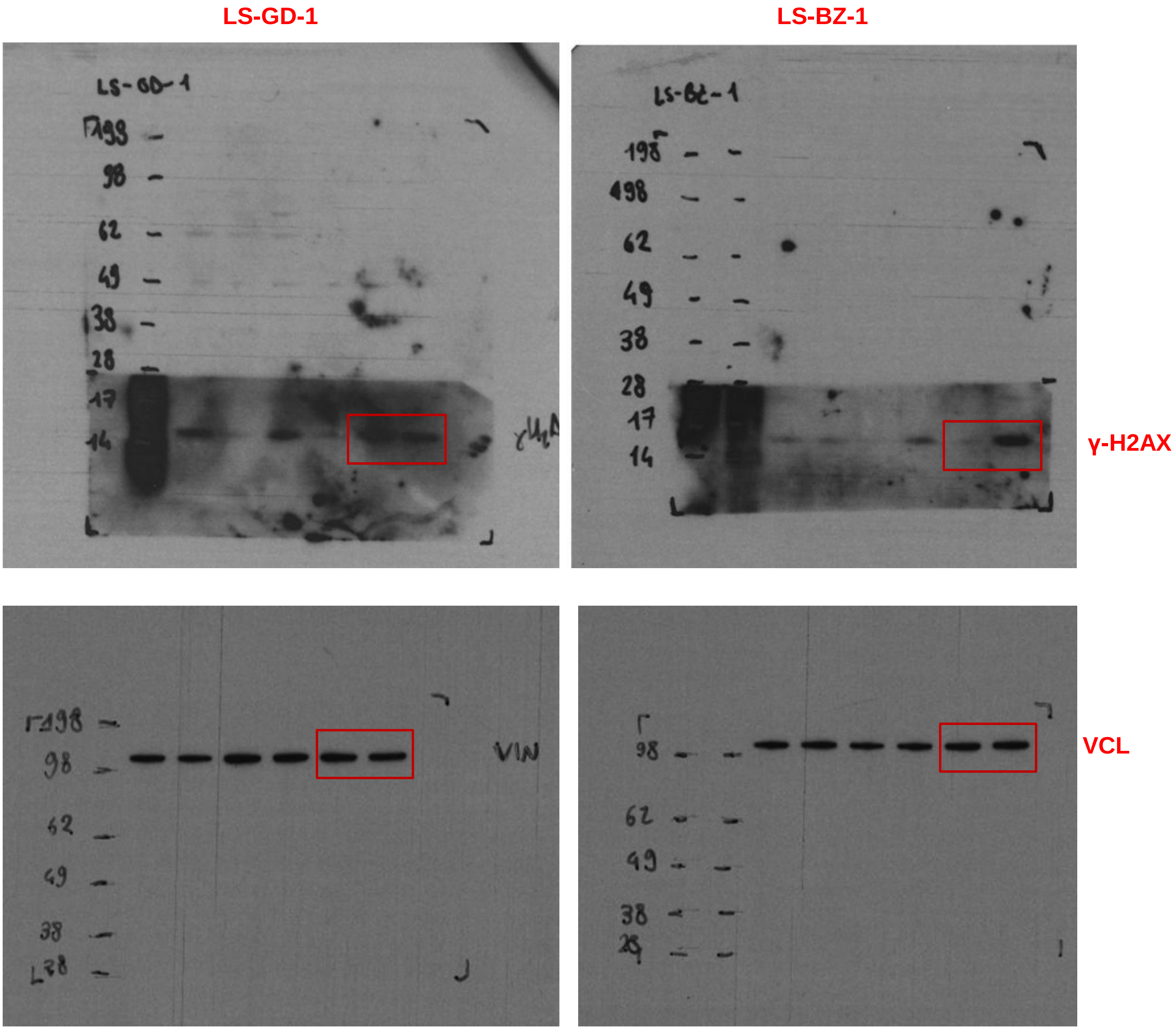

Figure S7. Full Western blot of Figure 3 (B).

First replicate

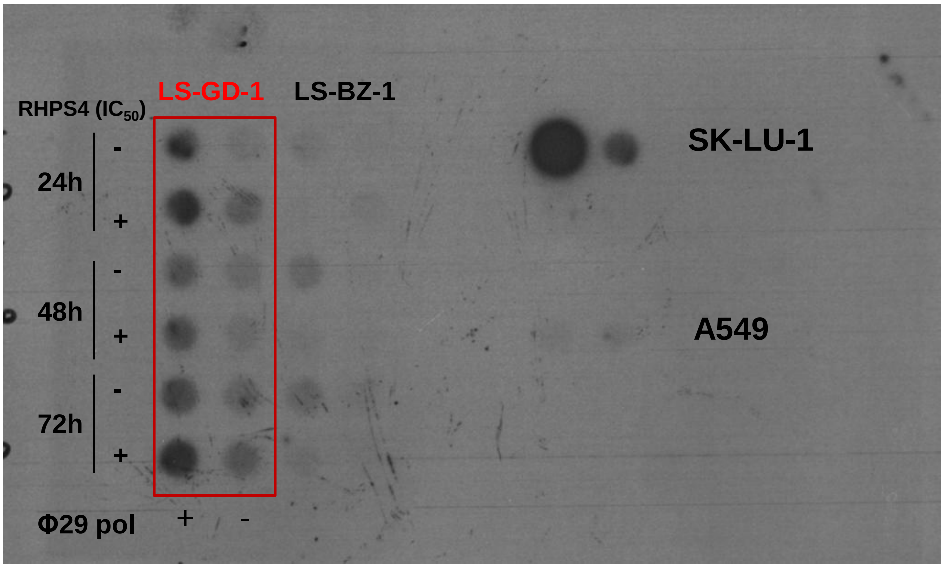

Second replicate

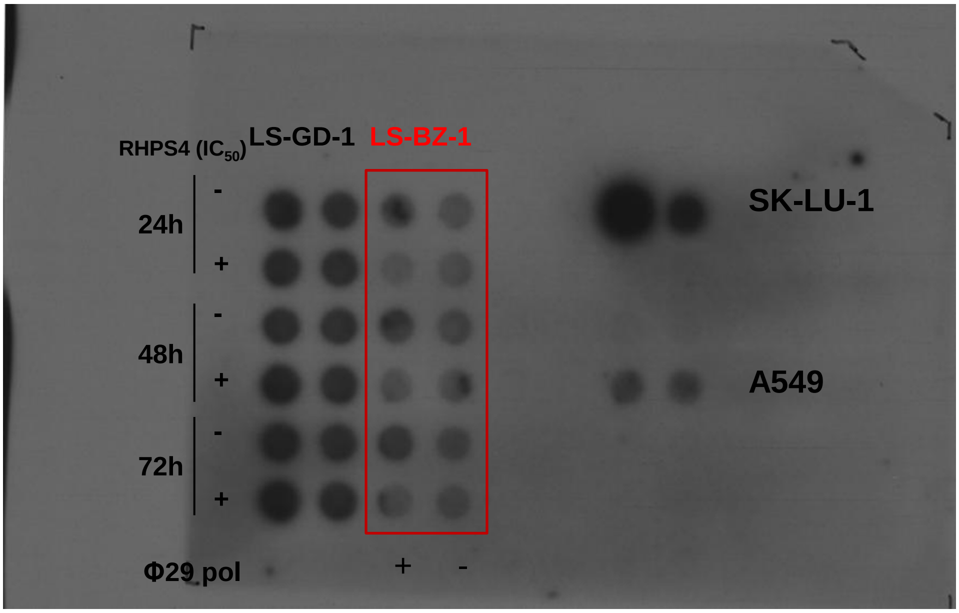

Figure S8. Full Dot blot of Figure 3 (D).

First replicate

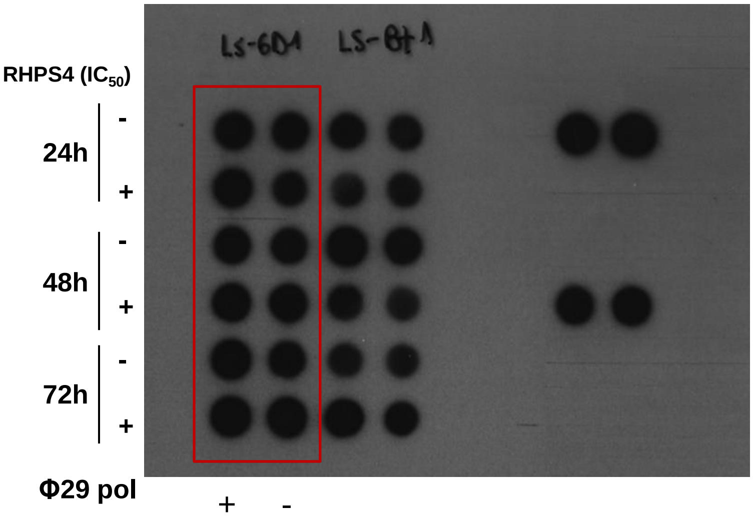

Second replicate

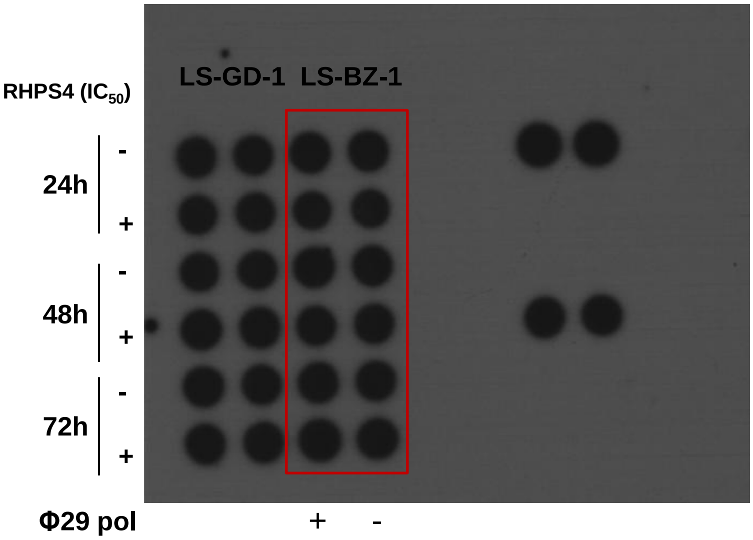

Figure S9. Full Dot blot of Figure S2.

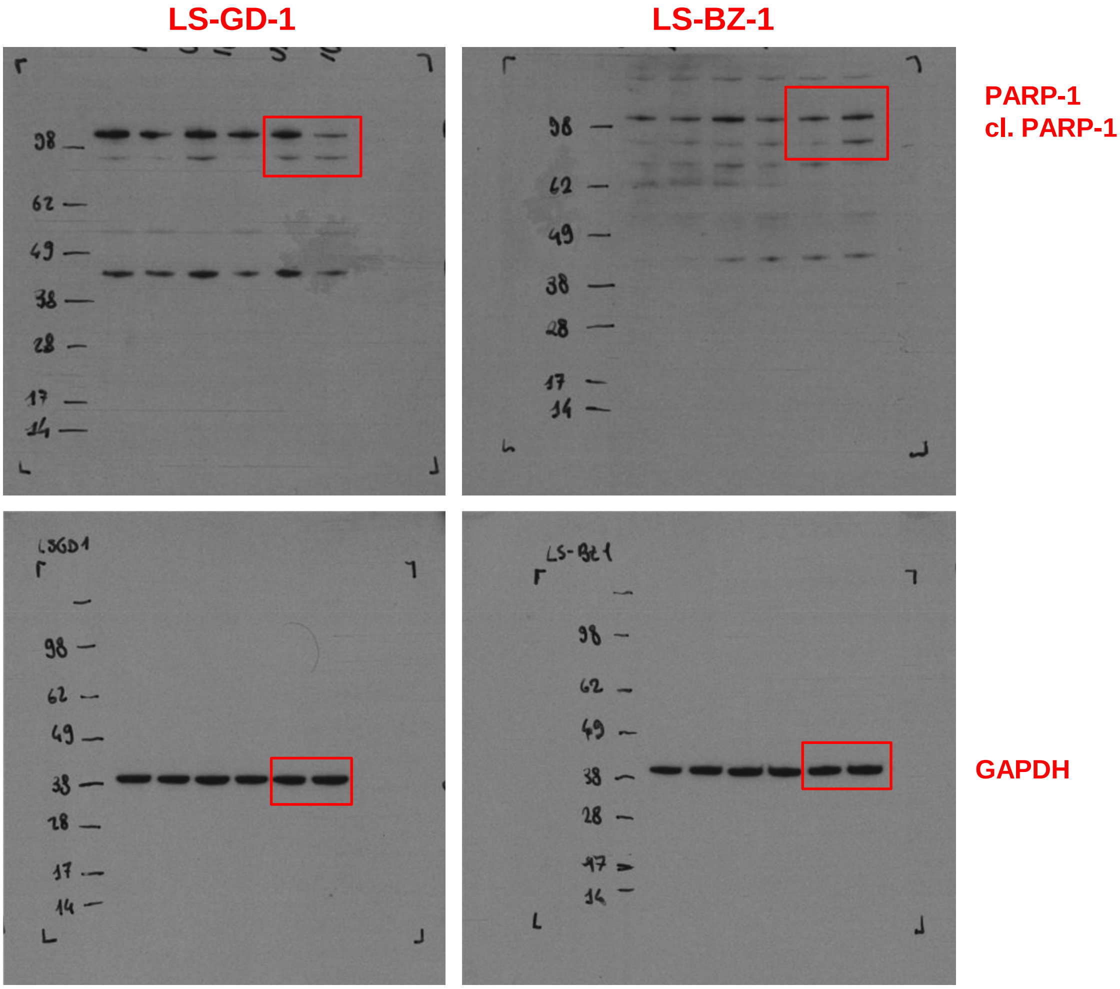

Figure S10. Full Western blot of Figure 4 (D).

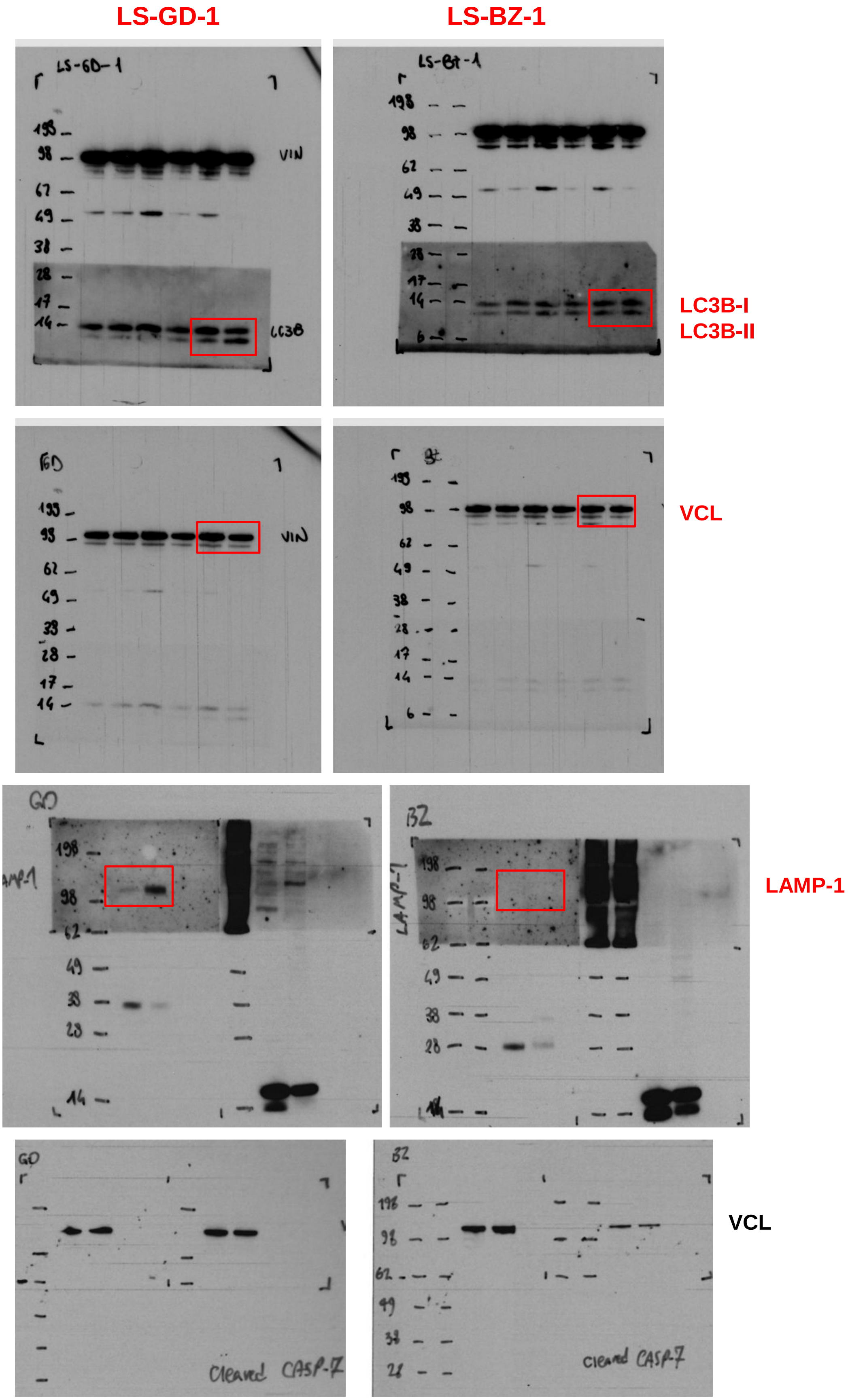
LS-GD-1
LS-BZ-1
LC3B-I
LC3B-II
VCL
LAMP-1
VCL
